# Imbalance of B-Cell Subpopulations in the Microenvironment of Sarcoidosis or Lung Cancer

**DOI:** 10.3390/cells13151274

**Published:** 2024-07-29

**Authors:** Agata Raniszewska, Iwona Kwiecień, Elżbieta Rutkowska, Joanna Bednarek, Rafał Sokołowski, Piotr Miklusz, Piotr Rzepecki, Karina Jahnz-Różyk

**Affiliations:** 1 Laboratory of Hematology and Flow Cytometry, Department of Internal Medicine and Hematology, Military Institute of Medicine-National Research Institute, 04-141 Warsaw, Poland; ikwiecien@wim.mil.pl (I.K.); erutkowska@wim.mil.pl (E.R.); 2 Department of Internal Medicine, Pulmonology, Allergology and Clinical Immunology, Military Institute of Medicine, 04-141 Warsaw, Poland; jbednarek@wim.mil.pl (J.B.); rsokolowski@wim.mil.pl (R.S.); pmiklusz@wim.mil.pl (P.M.); kjrozyk@wim.mil.pl (K.J.-R.); 3 Department of Internal Medicine and Hematology, Military Institute of Medicine-National Research Institute, 04-141 Warsaw, Poland; przepecki@wim.mil.pl

**Keywords:** sarcoidosis, lung cancer, lymph nodes, B cells, EBUS-TBNA, class-switched memory B cells, CD21low cells

## Abstract

Although the role of T lymphocytes in sarcoidosis (SA) and lung cancer (LC) is quite well reported, the occurrence of B cells in disease microenvironments may suggest their potential role as natural modifiers of the immune response. The aim of this study was to investigate the B-cell profile and lymphocyte-related hematological parameters between patients with SA, LC and healthy controls (HCs). The cells were assessed by flow cytometry and a hematological analyzer in peripheral blood (PB) and material from lymph nodes (LNs) obtained by the EBUS/TBNA method. We showed that in SA patients, there were higher percentages of naïve B and CD21low B cells and a lower percentage of class-switched memory B cells than LC patients in LNs. We observed a higher median proportion of non-switched memory and transitional B cells in the PB of SA patients than in LC patients. We noticed the lowest median proportion of class-switched memory B cells in the PB from SA patients. LC patients had a higher percentage of RE-LYMP and AS-LYMP than SA patients. Our study presented a different profile of B-cell subpopulations in SA and LC patients, distinguishing dominant subpopulations, and showed the relocation from distant compartments of the circulation to the disease microenvironment, thus emphasizing their role.

## 1. Introduction

Sarcoidosis (SA) is a systemic inflammatory disease of unknown cause. The diagnosis of SA is based on a clinical examination, imaging tests and histopathological examination, which should demonstrate the presence of inflammatory infiltrates and accumulated nodules, called non-caseous granulomas [1,2]. It is necessary to exclude other granulomatous diseases and local sarcoid reaction. The formation of sarcoid granuloma involves many immunological mechanisms. The unknown antigen is phagocytosed by macrophages or dendritic cells and then presented via the human leukocyte antigen (HLA) system to T-cell receptors (mainly CD4+ T cells). Cells accumulate as a result of their redistribution from the blood and local multiplication. Sarcoid granuloma consists of epithelioid and giant cells surrounded by lymphocytes and fibroblasts [3,4]. However, CD4+ T cells predominate; there are also single CD8+ T lymphocytes and B lymphocytes [2,5]. The accumulation of B lymphocytes in granulomatous lesions suggests a probable role in the type of humoral immune reactions in the pathogenesis of SA [6]. 

The lung cancer (LC) microenvironment consist of phenotypically and functionally distinct components, including fibroblasts, endothelial cells, stem cells and infiltrating immune cells participating in the development and progression of cancer and, consequently, the patient’s prognosis. The latest research focuses on the use of immune-checkpoint inhibitors related to T lymphocytes, combination immunotherapy, tumor-infiltrating lymphocyte (TIL) therapy, T-cell receptor (TCR) engineering, chimeric antigen receptor T-cell (CAR-T) cell therapy and lung cancer targeting tumor-specific vaccines [7,8]. Despite progress in the treatment of LC, which continues to be refined, ranging from chemotherapy to molecular targets to the latest immunotherapy methods, LC remains a global problem [9]. LC remains the most common malignant tumor in the world—2.5 million new cases were recorded in 2022, which constituted 12.4% of the total number of new cases of all cancers [10]. The lack of tumor-specific antigens, the large influence of the tumor microenvironment (TME) and toxicity make the above treatment options ineffective in some patients, as statistics show.

Therefore, it seems attractive to search and try to understand how other cell subpopulations participate in a specific disease microenvironment. In recent years, research emphasizing the role of B lymphocytes has become increasingly popular [11]. Emerging reports indicate that B lymphocytes play a role, not acting solely as a passive immune cell, in the TME. Depending on the environment, they may have anti-cancer or pro-cancer effects [12].

**Figure 1:**
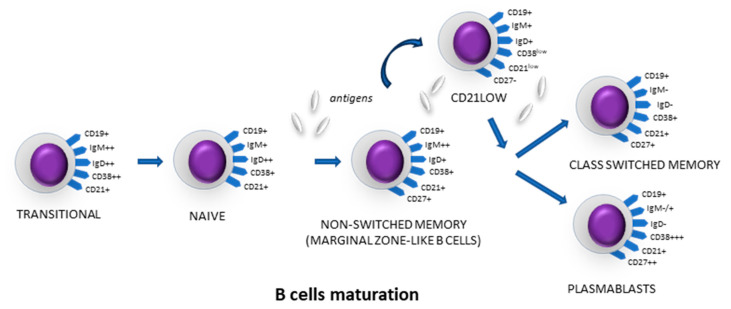
Antigen expression in B-cell subsets depending on maturation.

Understanding the mechanisms by which B-cell subtypes participate in creating the microenvironment of various diseases may redefine therapeutic targets, designate new points of immunotherapeutic strategies and act as a marker of immune checkpoints. After leaving the bone marrow, B lymphocytes become transitional cells and, migrating to peripheral lymphoid organs, transform into naïve B lymphocytes [13]. For a humoral immune response to occur, B lymphocytes must be activated, allowing the production of antibodies and the formation of immunological memory. The activation process most often occurs in secondary lymphoid organs such as LNs and the spleen. After recognizing a foreign antigen, naïve B cells transform into memory cells with the ability to switch antibody classes, as well as antibody-secreting plasmablasts [14]. B-cell activation reduces the level of surface expression of the CD21 antigen. CD21low cells are activated and have properties that enable them to differentiate into plasma cells [15]. They can interact with other cells of both the innate and adaptive immune response, ultimately leading to the production of cytokines [16]. By assessing the expression level of antigens such as CD19, CD20, CD21, CD27 and CD38 using flow cytometry, it is possible to determine the stages of the maturation process of B lymphocytes from naïve cells to cells expressing immunoglobulins. Figure 1 shows a diagram of the maturation of a B lymphocyte with the surface expression of antigens.

In our previous studies, we confirmed the utility of flow cytometry in analyzing lymphocyte subsets in lymph node (LN) aspirates achieved by the EBUS-TBNA method in LC and SA patients [17,18,19]. However, although the function of T lymphocytes in LC and SA has been quite well described, B-cell function in the TME and sarcoidosis granuloma has not been studied in detail. The EBUS-TBNA method, which allows for the collection of material from a lymph node, is a routine diagnostic test for mediastinal lymphadenopathy in respiratory medicine. Importantly, the procedure is safe for the patient, usually performed without general anesthesia and well tolerated [20,21]. LN aspirates obtained during the EBUS/TBNA technique are a liquid and cell-rich material, which can be analyzed using flow cytometry and a hematological analyzer. Currently available hematology analyzers enable not only routine blood counts, but using new parameters they are able to assess the cellular composition of each liquid material, which may be useful in the initial assessment of inflammatory diseases. The activation status of lymphocytes can now be quantified. The combination of new hematological parameters: antibody-synthesizing lymphocytes (AS-LYMP) and reactive lymphocytes (RE-LYMP) parameters, provides additional information on cell activity in the innate and adaptive immune response. Activated cells not only have a different composition of membrane lipids, but also show greater activity in the cytoplasm by producing cytokines. Consequently, the fluorescence signal intensity of activated cells is greater than the fluorescence signal of resting cells. The values of these parameters depend on the type of inflammatory factor, exacerbation and stage of infection. By interpreting these two parameters, it can be indicated whether we are dealing with a cellular or humoral immune response [22,23]. Additionally, lymphocyte populations can be differentiated based on their functional properties, differences in internal structure, granulation intensity and the surface of the examined cells. These parameters can be measured using the lymphocyte position variables in the WDF scattergram: laterally scattered light intensity (LY-X), intensity of fluorescent light (LY-Y), intensity of frontally scattered light (LY-Z), laterally scattered light intensity; the width of dispersion of lymphocyte complexity (LY-WX) and the intensity of fluorescent light; and the width of dispersion of lymphocyte fluorescence (LY-WY) [24,25]. 

In this study, we deliberately selected two diseases occurring with enlarged LNs to examine the involvement of different B-cell subpopulations and to check whether the profile of leukocytes and mainly the maturation of B lymphocytes is different in two different diseases. The involvement of B lymphocytes in SA is not entirely clear, as well as in LC.

The aim of our work was to analyze the maturation profile of B lymphocytes and new available hematological parameters: RE-LYMP, AS-LYMP and parameters related to lymphocyte differentiation, in different disease microenvironments: SA and LC, and to analyze these differences in peripheral blood (PB) from patients with SA, LC and healthy volunteers (HCs).

## 2. Materials and Methods

### 2.1. Patients 

The study enrolled 34 patients with a confirmed diagnosis of the lung disease SA and 33 patients with primary LC.

Material for cytological and flow cytometric analysis was obtained using the EBUS-TBNA method. SA disease was verified by histopathological examination. The diffusing capacity for carbon monoxide (DLCO) parameter was estimated for each patient according to the American Thoracic Society/European Respiratory Society guidelines [26]. Additionally, in each patient during the diagnosis of SA a spirometry functional test was performed. Among the results of lung function tests, forced vital capacity (FVC) and forced expiratory volume in the first second (FEV1) were reported.

The current histological classification [27] and the eighth edition of the TNM classification of lung cancer were used to confirm LC cases [28] (the beginning of the study was earlier than the 9th edition of the TNM lung cancer classification [29]). Histological examination revealed the following: small cell lung cancer (SCLC) 60.6% and non-small cell lung cancer (NSCLC) 39.4%; squamous cell lung cancer (SQCLC), n = 4 (30.8%); adenocarcinoma (ADC), n = 7 (30.8%); not otherwise specified (NOS), n = 1 (7.7%); and large cell carcinoma (LC), n =1 (7.7%). The small number of patients made it impossible to create groups with different types of disease and different stages of the disease.

Each patient gave written informed consent (Medical Chamber in Warsaw: KB/1441/23/ Military Institute of Medicine Ethics Committee: 25/WIM/2018) before proceeding with the medical procedure for EBUS/TBNA and PB collection. The general characteristics of patients are summarized in Table 1.

### 2.2. Materials: Lymph Node Aspirates and Peripheral Blood 

Material from the group of nodes LN 4, 7, 10 and 11 was collected during the standard EBUS/TBNA diagnostic method in patients with SA and LC. An additional sample was intended for hematological and cytometric analysis. In total, 1 mL of LN fluid was collected into a test tube with K_2_EDTA and diluted in 0.9% NaCl. Only positive lymph nodes for both LC and SA were included in the study. 

A total of 2 mL of PB was collected into test tubes containing K_2_EDTA.

### 2.3. Methods

#### 2.3.1. Hematological Parameters 

PB and LN aspirates were examined using the Sysmex XN-1500 analyzer (Sysmex Corp., Kobe, Japan). The instrument enables the analysis of PB samples, as well as the standardized measurement of body cavity fluids. The parameters measured by the hematological analyzer are presented in Table 2.

#### 2.3.2. Flow Cytometry Analysis

Cell subpopulations were assessed by multiparameter flow cytometry with a panel of monoclonal antibodies, performed using DxFLEX flow cytometry (Beckman Coulter Company, Marseille Cedex 9, France).

**Figure 2:**
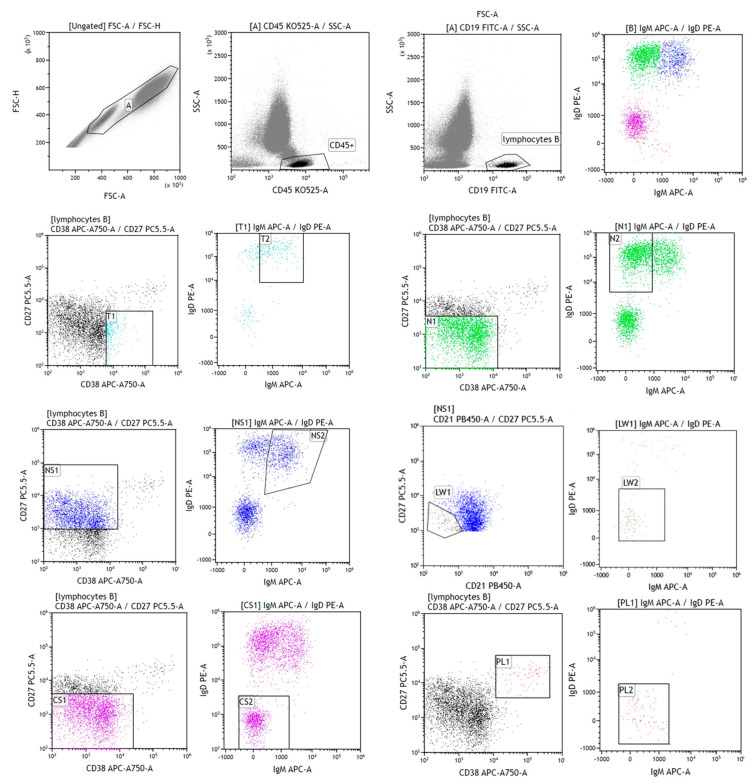
Lymphocyte maturation gating strategy in peripheral blood of selected patient with sarcoidosis. FSC-A vs. FSC-H plot: Gating the cells, eliminating clumps (greater FSC-A relative to FSC-H) and debris (very low FSC). CD45 vs. SSC-A plot: selection of lymphocytes based on their SSC/CD45 properties: low SSC and high CD45 (black cells). CD19 vs. SSC-A plot: selection of lymphocytes B based on their SSC/CD19 properties: low SSC and high CD19. IgD vs. IgM plot: All analyzed B-cell populations presented on one plot. The strategy for separating individual subsets is below: T1, T2—transitional B cells (turquoise cells), N1, N2—naïve B cells (green cells), NS1, NS2—non-switched memory B cells (blue cells), LW1, LW2—CD21low (dark green cells), CS1, CS2—class-switched memory B cells (purple cells), and PB1, PB2—plasmablasts (red cells). Populations of maturing B lymphocytes were marked based on the expression of the CD27/CD38 and IgD/IgM antigens.

For the assessment of B lymphocytes, the CD19-PE-Cy7 antibody (catalogue number: IM3628, clone number: J4.119, Beckman Coulter) was used. Additionally, the HLA-DR-V450 (catalogue number: 655874, clone number: L243, BD Biosciences) and CD45-V500 (catalogue number: 655873, clone number: 2D1 BD Biosciences) antibodies were used. Moreover, among B lymphocytes, we distinguished B-cell subpopulations using the following antibodies: IgD PE (catalogue number: 555779, clone number: IA6-2, BD Biosciences), CD27 PerCP-Cy5-5 (catalogue number: 656643, clone number: L128, BD Biosciences), IgM APC (catalogue number: 551062, clone number: G20-127, BD Biosciences), CD38 APC-H7 (catalogue number: 656646, clone number: HB7, BD Biosciences) and CD21 V450 (catalogue number: 658169, clone number: B-ly4, BD Biosciences). According to the EuroFlow consortium [30] (Figure 2), there are several categories:

- transitional B cells: IgM++ IgD++ CD38++ CD27- CD21+;

- naïve B cells: IgM+ IgD++ CD38+ CD27- CD21+;

- non-switched memory B cells (marginal zone-like B cells): IgM++ IgD+ CD38+ CD27+ CD21+;

- class-switched memory B cells: IgM- IgD- CD38+ CD27+ CD21+; 

- CD21low B cells: IgM+ IgD+ CD38low CD27- CD21low;

- plasmablasts: IgM-/+ IgD- CD38+++ C27++ CD21+. 

Additionally, in both LN aspirates and PB materials, basic leukocyte subsets were also isolated: T lymphocytes—CD4 and CD8 cells; NK cells, neutrophils and monocytes; and also tumor cells in LN aspirates from LC patients. Surface staining with fluorescently labeled antibodies was used: Ki-67-FITC (catalogue number: F7268, clone number: MIB-1, Dako) CD33-PE (catalogue number: 345799, clone number: PE7.6, BD Biosciences), CD3-PC-5.5 (catalogue number: B49203, clone number: UCHT1, Beckman Coulter), CD8-APC (catalogue number: IM2469, clone number: B9.11, Beckman Coulter), CD326 (Ep-CAM)- AF700 (catalog number: 324244, clone number: 9C4), CD16-APC-H7 (catalogue number: 560195, clone number: 3G8, BD Biosciences), HLA-DR-V450 (catalogue number: 655874, clone number: L243, BD Biosciences), CD45-V500 (catalogue number: 655873, clone number: 2D1, BD Biosciences) and CD4-BV650 (catalog number: 300536, clone number: RPA-T4, BioLegend). Cells were distinguished based on their antigenic properties: T lymphocytes (CD45+ CD3+ SSC-A+dim), CD4+ T lymphocytes (CD45+ CD3+ SSC-A+dim CD4+), CD8+ T lymphocytes (CD45+ CD3+ SSC-A+dim CD8+), NK cells (CD45+ CD3- SSC+dim CD16+), neutrophils (CD45+ CD3- SSC-A+bright CD16+), monocytes (CD45+ CD33+ HLA-DR+ SSC-A+) and tumor cells (CD45- Ep-CAM+ Ki-67+ SSC-A+bright). 

#### 2.3.3. Flow Cytometry Analysis

*p* < *0.05* was considered statistically significant. All statistical analyses were conducted using Statistica 13.0 software (TIBCO Software, Palo Alto, CA, USA) and the Prism GraphPad graphical and statistical program (version 7, GraphPad Software, La Jolla, CA, USA). The GraphPad program was used to graphically process the results. Results are expressed as means and SD, medians with interquartile range (Q1–Q3). A non-parametric test comparing the ranks of two independent samples (Mann–Whitney U test) and non-parametric tests for three groups (Kruskal–Wallis with the post-hoc Wilcoxon’s signed rank test) were used. 

## 3. Results

To assess the subpopulation of B lymphocytes, we analyzed the LN aspirates by the flow cytometry method Additionally, the PB from the same patients and from HCs was analyzed. Patients were also examined for new hematological parameters (for a description of the new hematological lymphocytes parameters, see Table 2 in the Section 2). Table 1 presented the clinical characteristics of the study groups. The study groups consisted of patients with SA (n = 34) and LC (n = 33) compared to the HC group (n = 20). The mean percentages of the primary leukocyte and lymphocyte subtypes are shown in Table 3.

### 3.1. Leukocyte Subpopulations in Lymph Node Aspirates 

In patients with SA, lymphocytes dominated in the LNs. However, in patients with LC, we noticed that the dominant cell population in the LNs was cancer cells (very numerous, approximately: median 88.5%). To reliably compare the percentage of lymphocyte subpopulations, we analyzed the population in the lymphocytic region.

In the SA patients, we noticed a significantly higher median proportion of CD4+ T lymphocytes and B lymphocytes than in LC patients. The CD8+ T lymphocyte median proportion was higher in patients with lung cancer than in sarcoidosis patients. We did not observe differences in the proportion of all lymphocytes and natural killer (NK) cells between patients with SA and LC patients (Table 3). 

### 3.2. B-Cell Maturation Subsets in Lymph Node Aspirates 

**Figure 3:**
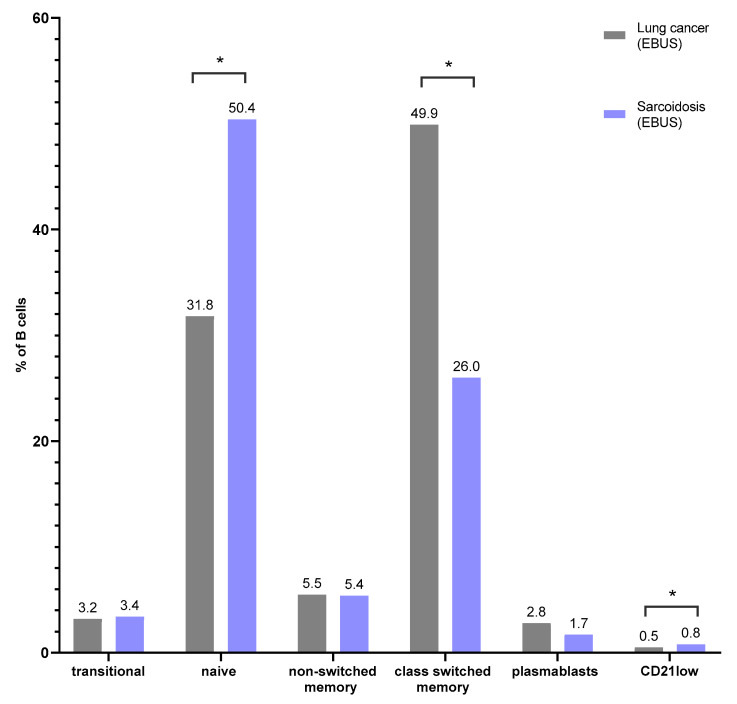
The differences in the median proportion of B-lymphocyte subsets: transitional B cells, naïve B cells, non-switched memory B cells, class-switched memory B cells, plasmablasts and CD21low cells in lymph node aspirates (LNs) between patients with lung cancer (LC) and sarcoidosis (SA). Graphs show the median values (* *p < 0.05*).

There was a higher median proportion of naïve B cells in SA patients than in LC (respectively, 50.4% vs. 31.8%, *p* = 0.0033). In this study, we observed a significantly higher median proportion of class-switched memory B cells in LC patients than in SA (49.9% vs. 26.0, *p* = 0.0019). When we analyzed the median proportion of CD21low B cells, we noticed a higher proportion in SA than in LC patients (respectively, 0.8% vs. 0.5%, *p* = 0.0377) (Figure 3). There were no differences in the proportion of transitional, non-switched memory B cells and plasmablasts in SA and LC patients (Table 4).

### 3.3. New Lymphocytes and Neutrophils Parameters in Lymph Node Aspirates 

We observed a significantly lower median proportion of the LY-X parameter (respectively, 81.7 vs. 85.6%, *p* < 0.0001) and a higher median proportion of the LY-Y parameter (respectively, 68.0 vs. 48.7%, *p* < 0.0001) in SA than in LC patients (Table 4). 

### 3.4. Leukocyte Subpopulations in Peripheral Blood 

We observed the significantly highest median proportion of white blood cell count (WBC) in LC patients than in SA patients and HCs (respectively, 8310 vs. 5730 vs. 5365, *p* = 0.0006). When we analyzed the leukocytes subpopulation, we observed a lower median proportion of lymphocytes, T lymphocytes, including CD4 T cells, CD8 T cells and NK cells in LC and SA patients than in HCs. We noticed a lower median proportion of B lymphocytes in LC patients than in HCs (respectively, 2.2% vs. 3.9%, *p* = 0.0065). There was a higher median proportion of neutrophils in LC patients than in SA patients and HCs (respectively, 77.3% vs. 69.0% vs. 47.1%, *p* < 0.0001). We observed a higher median proportion of monocytes in SA than in LC patients (respectively, 9.8 vs. 6.8%, *p* < 0.0046) (Table 5).

### 3.5. B-Cell Maturation Subsets in Peripheral Blood

**Figure 4:**
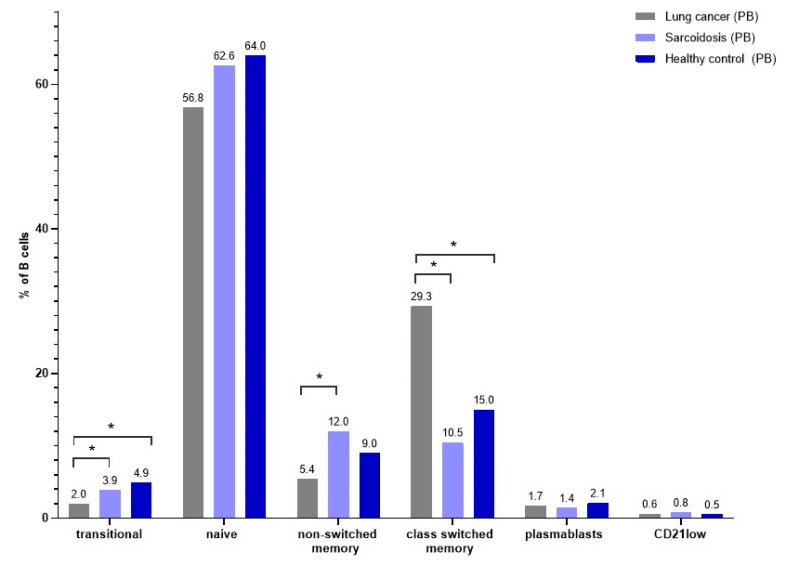
The differences in the median proportion of B-lymphocyte subsets: transitional B cells, naïve B cells, non-switched memory B cells, class-switched memory B cells, plasmablasts and CD21low cells in peripheral blood between patients with lung cancers (LC), patients with sarcoidosis (SA) and healthy control patients (HC). Graphs show the median values (* *p* < *0.05*).

There was a lower median proportion of transitional B cells in LC patients than in SA and in HC patients (respectively, 2.0% vs. 3.9% vs. 4.9%, *p* < 0.0001). In this study, we observed a significantly higher median proportion of class-switched memory B cells in LC patients than in SA and HC patients (respectively, 29.3% vs. 10.5% vs. 15.0%, *p* < 0.0001). Patients with SA had the lowest percentage of non-class-switched memory B cells, even lower than HCs. After examining the average CD21low proportion of B cells, we noticed a higher proportion in SA patients (respectively, 0.8% vs. 0.5%, *p* = 0.0377). We noticed a highest median proportion of non-switched memory B cells in PB from SA than LC and HC patients (respectively, 12.0% vs. 5.4% vs. 9.0%, *p* = 0.0002) (Figure 4). There were no differences in the proportion of naïve B cells, plasmablast and CD21low cells in the PB from SA, LC and HC patients (Table 6).

### 3.6. New Lymphocyte Parameters in Peripheral Blood 

**Figure 5:**
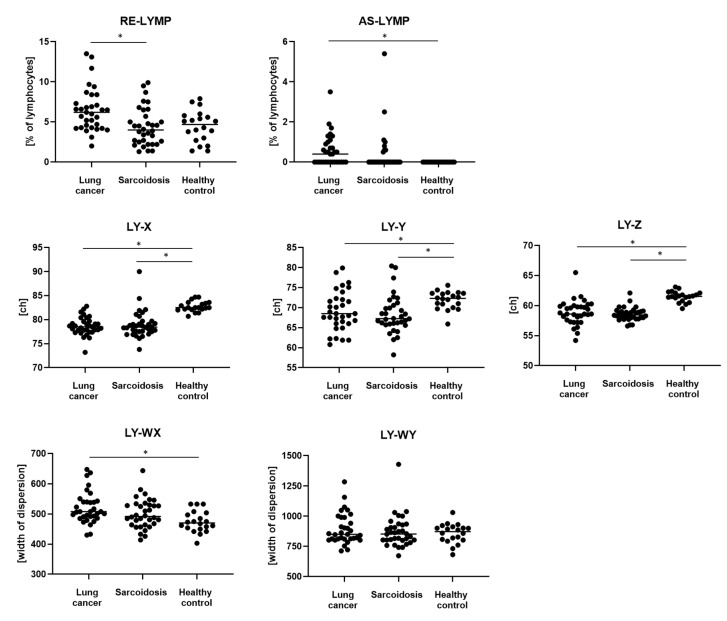
The differences in the proportion of lymphocytes and neutrophils Sysmex parameters between patients with lung cancer (LC), patients with sarcoidosis (SA) and healthy controls (HC). Data are expressed as the median. * indicates *p* is statistically significant. Abbreviations: AS-LYMP, antibody-synthesizing lymphocytes; CH, channel unity; LY-X, lymphocytes complexity; LY-Y, lymphocytes fluorescence; LY-Z, lymphocytes size; LY-WX, width of dispersion of lymphocytes complexity; LY-WY, width of dispersion of lymphocytes fluorescence; RE-LYMP, reactive lymphocytes.

We observed a significantly higher median proportion of RE-LYMP parameters in LC patient than in SA patients (respectively, 5.9% of lymphocytes vs. 4.0% of lymphocytes, *p* = 0.0316) and a higher median proportion of AS-LYMP parameter in LC patients than in HCs (respectively, 0.4% of lymphocytes vs. 0.0% of lymphocytes, *p* = 0.0032). We noticed a lower median proportion of LY-X, LY-Y and LY-Z parameters in SA patients (respectively, 78.4, 67.2 and 58.4) and LC patients (respectively, 78.5, 68.5 and 58.6) than in HCs (respectively, 82.5, 72.3 and 61.1). Statistically significant *p*-values were detailed for the above-mentioned parameters in Table 6. The LY-WX parameter was higher in LC patients than in HCs (respectively, 508.0 vs. 470.5, *p* = 0.0032), Table 6, Figure 5. 

## 4. Discussion

In both SA and LC disease, a characteristic microenvironment is created in the affected LNs. In our previous study, we confirmed that LN aspirates obtained during the EBUS/TBNA procedure could be useful in the description of the granuloma microenvironment [17]. Current research indicates that the mechanism of SA development involves immune cells (e.g., macrophages, dendritic cells, subtypes of T cells, etc.), which accumulate and are excessively activated in organs or tissues with antigens of their own or foreign origin [31]. Trying to explain these phenomena (and perhaps also the involvement of individual B-cell subtypes), we wanted to further understand the pathogenesis of SA. The role of B cells in the development of other granulomatous diseases has been pointed out. B lymphocytes are responsible for the pathological changes present in granulomatosis with polyangiitis, and therapy directed at these cells seems to be extremely effective [32]. In this study, the reference group for SA patients was intentionally composed of patients with LC, with a different immunological mechanism but with a course also affecting the LNs. We have extensively studied the LC microenvironment [33,34]. It is widely believed that cells of the TME play a significant pro- and anti-tumor role. We assessed the multifaceted effects of T cells, monocytes, macrophages and dendritic cells in LC [19,35,36,37,38,39,40,41]. However, although the role of the cells mentioned above in the TME is relatively well described, B-cells’ function remains unknown—B lymphocytes interact with T lymphocytes, both CD4+ and CD8+ cells, promoting anti-tumor immunity by presenting antigens and transmitting signals to activated lymphocytes [14]. The latest research on B lymphocytes shows that the role of these in LC may be very variable and flexible, both pro- and anti-cancer [42]. 

It seems interesting to assess how we can use knowledge about B-cell biology in the disease microenvironment along with other existing leukocyte subpopulations.

In the current study, the median percentage of CD8+ T cells was higher in patients with LC than in patients with SA. There were no differences in the proportion of total lymphocytes and NK cells between patients with LC and patients with SA. SA patients had a higher median percentage of CD4+ T cells and B cells than LC patients, but we observed interesting differences when assessing the B-cell maturation profile. After reviewing the literature, we conclude that this study is the first to describe B-cell subsets: naïve, non-switched memory, transitional, CD21low, class-switched memory B and plasmablasts, in EBUS-TBNA aspirates in SA and LC patients. 

When comparing SA patients with the group of LC patients, the dominant population among B lymphocytes in LC patients was class-switched B cells. The literature data showed that plasma cells and memory cells appeared to be a significant component of the TME [43]. Patel et al. evaluated B-cell subpopulations measured by mass cytometry assessment in LC and also demonstrated the presence of memory B cells at different stages of maturation: early memory B cells, fully affinity mature class-switched B cells and double class-switched negative memory B cells [43]. Hao et al. showed that in lung adenocarcinomas, memory B cells in tumor tissues were mainly class-switched and at a late germinal center stage [44]. The authors highlighted the potential impact of class-switched memory B cells on tumor immunity. Class-switched memory B cells were particularly significant in predicting outcomes. Patients with higher B-cell infiltrating tumors showed improved disease-free survival [45]. 

On the other hand, we found that SA patients had a lower percentage of class-switched-memory B cells in LN aspirates than LC patients. There are not many studies on the distribution of B-cell subpopulations in sarcoidosis granulomas. Kamphuis et al. confirmed the involvement of B lymphocytes in the pathogenesis of SA, showing their significant population surrounding the forming granulomas [46]. Lee et al. and Kudryavtsev et al. found by immunohistochemistry that B lymphocytes accumulated in granulomas in patients with pulmonary SA [47,48]. Despite reports describing the accumulation of B lymphocytes around granulomas, the role these cells may play in the development of SA has not been clarified.

Interestingly, in our study the percentage of class-switched-memory B cells in the PB was lower than in HCs. Kudryavtsev et al. performed an analysis of immune cell subsets in the PB of SA patients by flow cytometry [47]. Their results confirmed a disruption in the maturation of B-lymphocyte subsets and showed the role of T-helper cells in regulating the differentiation of B lymphocytes. There was an increase in the number of antibody-producing B lymphocytes and a decrease in the number of memory B lymphocytes. Similar disturbances in peripheral B-cell maturation in patients with SA with an increased numbers of transitional B cells and decreased numbers of class-switched memory B cells have been observed in patients with granulomatous common variable immunodeficiency (CVID) [49,50]. 

In our study, the predominant population in the LNs of SA patients was naïve cells. Naïve B cells were not stimulated with antigen and their increased number indicates the lack of an immunological reaction [51]. The predominance of naïve B lymphocytes and the low percentage of class-switched cells in LN aspirates in SA patients may indicate an inhibition of the immune response. There are no sufficient data describing the role of naïve B cells in the pathogenesis of SA. In line with our study, there are reports confirming the increased levels of naïve B cells in the PB of SA patients [6,52]. In autoimmune diseases manifesting as skin lesions, such as systemic sclerosis, systemic lupus erythematosus and psoriasis, the presence of significant numbers of naïve B cells in the skin has been described [53,54,55]. Their presence may indicate immunological tolerance in the course of the disease. This is referred to as peripheral tolerance described in primary immunodeficiencies and some autoimmune diseases [56,57]. Altogether, it might be suggested that a similar state of tolerance may occur during the development of SA, unlike LC where the previously mentioned class-switched memory B cells dominate, indicating the activation of LNs.

Transitional B cells maturing in the spleen overcome the negative selection caused by reactivity with self-antigens. This process is an important control point for negative selection, and the number of transitional cells in the periphery indicates the degree of intensity and correctness of this process [58,59]. In our study, SA patients had a higher percentage of transitional B cells than LC patients. A similar arrangement of B-lymphocyte subpopulations has been observed in a study by Kamphuis et al. [46] and with other studies, which compared the distribution of B-lymphocyte subtypes in the PB of SA patients and HCs [6,47,60]. In the above works, the role of transitional lymphocytes is emphasized. Although they are considered one of the regulatory B-cell subpopulations in healthy individuals, their frequency may be altered in individuals with autoimmune diseases [5]. Additionally, transitional B cells regulate the proliferation and differentiation of CD4+ T cells into effector T (Th) cells by releasing IL-10 [5,61]. This is quite the opposite of our results. In our study, SA patients had a lower (but not significantly) percentage of transitional B cells than HCs. 

Moreover, our data clearly revealed that the subpopulation of CD21low is increased in the LNs of SA compared to LC patients. CD21low cells are a subset of B cells that are increased in immune-related conditions, including common variable immunodeficiency, autoimmunity and chronic infectious diseases [62,63,64,65]. However, CD21low B cells exhibit an activated state, making them prone to co-stimulate T cells at sites of inflammation [66]. There are no studies assessing CD21low cells in sarcoidosis. Numerous CD21low B cells have been described in NSCLC tissue [67]. Also, CD21low B cells in the PB have been described to correlate with a poor response to checkpoint inhibitors in NSCLC [68]. CD21 cells have been observed in the PB in the case of viral infections such as HIV, SARS-Cov-2 or HCV, as well as chronic diseases such as malaria. Despite the higher amount of these cells, an exhausted phenotype was expressed as a result of constant exposure to the antigen [69,70,71,72]. In our study, we did not examine the activation state of CD21low B cells, but this may be the focus of further investigation. Taking into account that such differences do not belong to the PB, it can be suspected that in SA patients CD21low cells are responsible for reactions specifically in the disease microenvironment.

The activation of memory B cells with a specific antigen causes the formation of plasmablasts that circulate between the bone marrow and the PB. They are the most important source of antibodies with a high affinity for the antigen. AS-LYMP represents the activated B lymphocytes (plasma cells) responsible for antibody production. AS-LYMP cells are part of the RE-LYMP lymphocyte population [22]. In this study, SA patients showed lower percentages of RE-LYMP and AS-LYMP cells than LC patients. Elevated RE-LYMP levels can occur due to various factors, including inflammation, infections or autoimmune conditions. In cancer, increased RE-LYMP and AS-LYMP may be associated with the body’s response to tumor antigens or the TME. Monitoring AS-LYMP levels can provide insights into the immune system’s activity during cancer progression. Some work shows that plasma cells, which until recently were thought to play an important role in the development of antitumor responses, play a crucial function in the response to checkpoint blockade in LC patients [73,74]. However, RE-LYMP and AS-LYMP alone are not specific to cancer diagnosis; they require correlation with other clinical findings and tests. There were no statistically significant differences between RE-LYMP and AS-LYMP between SA patients and HCs. In our previous study, we confirmed the utility of RE-LYMP in the diagnosis of COVID-19 patients [75]. The RE-LYMP parameter was significantly higher in COVID-19 patients than in HCs. Comparing both studies, similar relationships between LC patients and COVID patients can be observed. It can be concluded that in the course of LC and COVID, we are dealing with a sudden inflammation rather than a chronic one as in the case of SA.

Finally, we revealed some significant differences in the lymphocyte Sysmex parameters. Both SA and LC patients had lower levels of the LY-X, LY-Y and LY-Z parameters than HCs. These parameters reflect to lymphocyte complexity, fluorescence and size, respectively. Some studies demonstrated that the LY-X, LY-Y and LY-Z lymphocyte parameters are useful for detecting morphological changes in lymphocytes [24]. The Sysmex analyzer proved beneficial in automatically differentiating neoplastic and reactive lymphocytosis. These parameters reflect activated lymphocytes which are larger than resting ones and have a modified shape of the nucleus with an irregular cell outline [76]. Interestingly, in our study, patients with the disease showed lower differentiation, size and fluorescence than healthy patients when the parameters were examined in the PB. However, interesting differences were observed in the LNs of patients with SA and LC. Here, lymphocytes from SA patients showed higher fluorescence but lower complexity than those from LC patients. This observation requires further research and correlation with other lymphocyte subpopulations, but due to the simplicity of method and its cost it may be an interesting parameter in the future. It should be emphasized, however, that the Sysmex parameters indicated significant differences in lymphocytes in the microenvironment of SA and LC and allowed us to distinguish the disease state from health in the PB. 

We acknowledge that this study is not without limitations. The main limitation is the size of the study groups, both LC and SA, which does not allow the analysis of patients with different LC subtypes and SA phenotypes. It is also known that SCLC and NSCLC have slightly different biological mechanisms of lymph node invasion and systemic impact [77,78], which we were unable to assess due to the size of the group. It should also be noted that the HC group is unbalanced (in terms of gender), which may also be a limitation of this study. However, to our knowledge, this is the first study using diseased LNs in which B-cell subsets in the LC and SA were assessed using applied flow cytometry techniques and a hematological instrument. The study demonstrates differences between LC, SA and HC patients, and as such provides a background for future research.

To summarize our studies, we showed a different profile of B-lymphocyte subpopulations in patients with SA and LC, indicating the dominant ones. The predominance of naïve B cells and lower percentage of class-switched B cells in SA patients than in LC patients may indicate differences in the inflammatory response. To summarize our observations, it seems that SA is a chronic disease where a certain type of immunological tolerance may develop, while the course of LC focuses on full activation of the inflammatory process.

## 5. Conclusions

The conducted research shows that there are significant differences in the composition of B lymphocytes and value of hematological parameters connected with B lymphocytes in SA and LC. Our findings show that the disease microenvironment has a strong influence on the dynamics of the B-cell profile. These differences were more visible in the examined aspirates from LNs than in PB. Our results further reinforce the phenomenon of B-cell relocation from distant compartments of the circulation to the disease microenvironment, thus highlighting the role of dominant subpopulations in creating a specific tolerance in SA or inflammation in LC.

## Figures and Tables

**Table 1:** Patient characteristics.

	SA	LC	HC
Number of patients	34	33	20
Sex F/M (n)	9/25	19/14	19/1
Age (mean ± SD years)	42.5 ± 13.0	67.0 ± 8.5	51.5 ± 9.7
LN 4/7/10/11/ *	2/16/1/15/0	15/6/1/5/6	n/a
Stage I/II/III/IV	11/23/ n/a /n/a	2/4/17/10	n/a
**SA patients characteristic**			
BMI (mean ± SD)		29.6 ± 5.0	
DLCO (>80%/<80%)		17/17	
DLCO > 80% (mean ± SD)		94.2 ± 7.6	
DLCO < 80% (mean ± SD)		70.6 ± 7.0	
FVC women [L]		3.10 ± 0.42	
FVC men [L]		4.22 ± 0.63	
FEV1 women [L]		2.50 ± 0.31	
FEV1 men [L]		3.10 ± 0.45	
FEV1/FVC		82.70 ± 6.50	

Abbreviations: HC—healthy control, LC—lung cancer; SA—sarcoidosis, DLCO—diffusing capacity for carbon monoxide, FVC—forced vital capacity; FEV1—forced expiratory volume in 1 s; *—tumor mass; n/a—not applicable.

**Table 2:** The parameters measured by hematological analyzer in peripheral blood (PB) and lymph nodes (LNs) samples.

Parameter	Parameter Description
AS-LYMP	quantifies the activated B lymphocytes that synthesize antibodies.
RE-LYMP	all activated lymphocytes with a higher level of fluorescence than resting lymphocytes
LY-X	laterally scattered light intensity; lymphocyte complexity
LY-Y	intensity of fluorescent light; reflects lymphocyte fluorescence
LY-Z	intensity of frontally scattered light; reflects lymphocyte size
LY-WX	laterally scattered light intensity; width of dispersion of lymphocyte complexity
LY-WY	intensity of fluorescent light; width of dispersion of lymphocyte fluorescence

**Table 3:** Differences in the proportion of leukocytes in lymph node aspirates (LNs) between patients with lung cancer (LC) and sarcoidosis (SA). Data are expressed as the median (Q1–Q3). * indicates *p* is statistically significant.

Leukocytes Subpopulation: [% of All Cells]	Lung Cancer Median (Q1–Q3)	Sarcoidosis Median (Q1–Q3)	* p < *0.05* Mann–Whitney U Test)
Lymphocytes	6.0 (1.5–8.4)	88.6 (82.1–98.3)	* p < *0.0001*
Lymphocytes T	3.4 (0.9–6.4)	50.2 (39.4–63.5)	* p < *0.0001*
CD4	1.4 (0.5–4.0)	37.0 (27.1–45.7)	* p < *0.0001*
CD8	1.4 (0.4–3.7)	12.9 (8.8–16.2)	* p < *0.0001*
Ratio CD4/CD8	1.2 (1.0–1.8)	3.0 (2.2–4.6)	* p < *0.0001*
Lymphocytes B	0.6 (0.3–1.5)	27.5 (17.6–43.3)	* p < *0.0001*
NK cells	0.2 (0.0–0.8)	1.4 (0.0–2.8)	* p = *0.0073*
Neutrophils	2.9 (0.0–14.2)	4.2 (0.0–13.4)	*p* = 0.7993
Monocytes	0.0 (0.0–0.0)	2.8 (0.0–9.5)	* p < *0.0001*
Tumor cells	88.5 (68.4–94.3)	-	-
**[% of all lymphocytes]**			
Lymphocytes T	75.6 (57.1–81.7)	70.0 (54.5–76.0)	*p* = 0.1084
CD4	37.9 (24.8–48.8)	49.8 (39.1–54.5)	* p = 0.0097
CD8	32.4 (24.7–42.1)	16.1 (11.1–23.6)	* p = 0.0002
Lymphocytes B	16.4 (11.9–26.1)	26.9 (21.1–45.5)	* p = 0.0039
NK cells	2.6 (0.0–12.0)	1.7 (0.1–3.8)	*p* = 0.3848

Abbreviations: WBC, white blood cells.

**Table 4:** Differences in the proportion of B-cell maturation subsets, CD21low cells and hematological parameter in lymph node aspirates (LNs) between patients with lung cancer (LC) and sarcoidosis (SA). Data are expressed as the median (Q1–Q3). * indicates *p* is statistically significant.

	Lung Cancer Median (Q1–Q3)	Sarcoidosis Median (Q1–Q3)	* p < *0.05* Mann–Whitney U Test)
**B-cell subpopulation:** **[% of B lymphocytes]**			
Naïve	31.8 (20.1–53.3)	50.4 (42.5–59.7)	* p = 0.0033
Non-switched memory	4.7 (3.1–7.0)	5.4 (2.5–14.8)	*p* = 0.3777
Class-switched memory	49.9 (26.3–62.7)	26.0 (15.0–39.6)	* p = 0.0019
Transitional	2.4 (1.1–4.7)	3.4 (1.2–8.7)	*p* = 0.1449
Plasmablast	2.8 (1.5–5.6)	1.7 (0.9–5.2)	*p* = 0.3533
CD21low cells	0.5 (0.0–0.9)	0.8 (0.2–1.6)	* p = 0.0377
**Hematological parameter:**			
LY-X [ch]	85.6 (83.0–96.6)	81.7 (79.9–82.8)	* p < *0.0001*
LY-Y [ch]	48.7 (44.2–54.4)	68.0 (65.5–73.1)	* p < *0.0001*

Abbreviations: ch, channel unity; LY-X, lymphocytes complexity, LY-Y, lymphocytes fluorescence.

**Table 5:** Differences in the proportion of leukocytes subsets in peripheral blood (PB) between patients with lung cancer (A), patients with sarcoidosis (B) and healthy patients (C). Data are expressed as the median (Q1–Q3). * indicates *p* is statistically significant.

		Lung Cancer (A) Median (Q1–Q3)	Sarcoidosis (B) Median (Q1–Q3)	Healthy Control (C) Median (Q1–Q3)	* p < *0.05* Group A-B-C ANOVA, Kruskal–Wallis	* p < *0.05* Group, in Groups Post-Hoc
WBC	[k/µL]	8310 (6500–11870)	5730 (4720–7460)	5365 (4695–6720)	* p = 0.0006	*A.-C. p* = *0.0041* *A.-B. p* = *0.0026*
Lymphocytes	[%]	14.8 (11.6–22.4)	25.5 (16.0–30.1)	41.3 (36.9–45.5)	* p < *0.0001*	*A.-C. p* < *0.0001* *A.-B. p* = *0.0174* *B.-C. p* = *0.0001*
T Lymphocytes	[%]	10.7 (6.7–17.1)	17.0 (10.0–20.4)	31.4 (26.4–35.4)	* p < *0.0001*	*A.-C. p* < *0.0001* *B.-C. p* < *0.0001*
CD4	[%]	4.9 (3.4–14.1)	10.1 (6.0–12.6)	19.7 (17.0–22.0)	* p < *0.0001*	*A.-C. p* = *0.0075* *B.-C. p* < *0.0001*
CD8	[%]	2.9 (1.4–3.8)	6.0 (4.2–8.5)	11.4 (8.3–14.7)	* p < *0.0001*	*A.-C. p* = *0.0014* *B.-C. p* = *0.0002*
Ratio CD4/CD8		2.4 (1.3–4.9)	1.5 (0.9–2.3)	1.7 (1.3–2.3)	*p* = 0.1944	-
B Lymphocytes	[%]	2.2 (1.2–2.9)	2.8 (1.3–3.7)	3.9 (2.7–5.6)	* p = 0.0089	*A.-C. p* = *0.0065*
NK cells	[%]	1.7 (0.8–3.5)	2.9 (1.4–3.7)	5.7 (2.7–7.7)	* p = 0.0002	*A.-C. p* = *0.0001* *B.-C. p* = *0.0134*
Neutrophils	[%]	77.3 (69.2–80.5)	69.0 (61.5–72.0)	47.1 (42.4–52.3)	* p < *0.0001*	*A.-C. p* < *0.0001* *A.-B. p* = *0.0191* *B.-C. p* < *0.0001*
Monocytes	[%]	6.8 (5.6–7.8)	9.8 (6.5–11.8)	7.9 (6.7–10.6)	* p = 0.0045	*A.-B. p* = *0.0046*

Abbreviations: WBC, white blood cells.

**Table 6:** Differences in the proportion of B-cell maturation subsets, CD21low cells and new hematological parameters in peripheral blood between patients with lung cancer (LC) (A), patients with sarcoidosis (SA) (B) and healthy patients (HC) (C). Data are expressed as the median (Q1–Q3). * indicates *p* is statistically significant.

Cells Subsets: [% of B Lymphocytes]	Lung Cancer (A) Median (Q1–Q3)	Sarcoidosis (B) Median (Q1–Q3)	Healthy Control (C) Median (Q1–Q3)	* *p* < 0.05 Group A-B-C ANOVA, Kruskal–Wallis	* *p* < 0.05 Group, in Groups Post-Hoc
**B-cell subsets:** **[% of B lymphocytes]**					
Naïve	56.8 (45.9–68.7)	62.6 (56.5–73.9)	64.0 (57.9–75.5)	*p* = 0.1216	-
Non-switched memory	5.4 (4.3–7.8)	12.0 (7.3–20.8)	9.0 (6.6–11.7)	* p = 0.0002	*A.-B. p = 0.0001*
Class-switched memory	29.3 (17.1–37.8)	10.5 (6.6–15.2)	15.0 (12.6–21.5)	* p < *0.0001*	*A.-B. p < 0.0001* *A.-C. p = 0.0103*
Transitional	2.0 (0.9–2.9)	3.9 (2.7–9.2)	4.9 (2.8–6.0)	* p < *0.0001*	*A.-B. p = 0.0003* *A.-C. p = 0.0004*
Plasmablast	1.7 (4.3–7.8)	1.4 (0.8–4.3)	2.1 (0.7–3.3)	*p* = 0.9488	-
CD21low	0.6 (0.3–1.4)	0.8 (0.3–1.7)	0.5 (0.3–0.7)	*p* = 0.4567	-
**Hematological parameters:**					
RE-LYMP [×10^3^/µL]	0.1 (0.1–0.2)	0.1 (0.0–0.1)	0.1 (0.0–0.1)	* p = 0.0017	*A.-B. p = 0.0013*
RE-LYMP [% of all cells]	1.9 (1.4–2.4)	0.9 (0.6–1.1)	1.0 (0.5–1.4)	* p < 0.0001	*A.-B. p = 0.0001* *A.-C. p = 0.0003*
RE-LYMP [% of lymphocytes]	5.9 (4.2–7.6)	4.0 (2.5–5.7)	4.4 (3.0–6.5)	* p = 0.0378	*A.-B. p = 0.0316*
AS-LYMP [×10^3^/µL]	0.0 (0.0–0.1)	0.0 (0.0–0.0)	0.0 (0.0–0.0)	* p = 0.0002	*A.-C. p = 0.0030*
AS-LYMP [% of all cells]	0.1 (0.0–0.2)	0.0 (0.0–0.0)	0.0 (0.0–0.0)	* p = 0.0003	*A.-C. p = 0.0038*
AS-LYMP [% of lymphocytes]	0.4 (0.0–0.9)	0.0 (0.0–0.0)	0.0 (0.0–0.0)	* p = 0.0002	*A.-C. p = 0.0032*
LY-X [ch]	78.5 (77.8–79.6)	78.4 (77.5–79.7)	82.5 (82.1–83.5)	* p < *0.0001*	*A.-C. p < 0.0001* *B.-C. p < 0.0001*
LY-Y [ch]	68.5 (66.0–72.0)	67.2 (65.9–70.6)	72.3 (70.4–73.6)	* p = 0.0028	*A.-C. p = 0.0283* *B.-C. p = 0.0022*
LY-Z [ch]	58.6( 57.6–59.9)	58.4 (58.0–59.0)	61.6 (61.0–62.1)	* p < 0.0001	*A.-C. p < 0.0001* *B.-C. p < 0.0001*
LY-WX	508.0 (491.0–540.0)	492.0 (464.0–530.0)	470.5 (452.0–500.5)	* p = 0.0032	*A.-C. p = 0.0021*
LY-WY	850.0 (813.0–990.0)	853.0 (804.0–928.0)	873.0 (806.0–907.5)	*p* = 0.7664	-

Abbreviations: AS-LYMP, antibody-synthesizing lymphocytes; ch, channel unity; LY-X, lymphocytes complexity; LY-Y, lymphocytes fluorescence; LY-Z, lymphocytes size; LY-WX, width of dispersion of lymphocytes complexity; LY-WY, width of dispersion of lymphocytes fluorescence; RE-LYMP, reactive lymphocytes.

## Data Availability

The data presented in this study are available in this article.
